# Attenuation of Human Respiratory Viruses by Synonymous Genome Recoding

**DOI:** 10.3389/fimmu.2019.01250

**Published:** 2019-06-04

**Authors:** Cyril Le Nouën, Peter L. Collins, Ursula J. Buchholz

**Affiliations:** RNA Viruses Section, LID, NIAID, NIH, Bethesda, MD, United States

**Keywords:** human respiratory virus, respiratory syncytial virus, influenza virus, vaccine, genome recoding, synonymous codon deoptimization, synthetic biology

## Abstract

Using computer algorithms and commercial DNA synthesis, one or more ORFs of a microbial pathogen such as a virus can be recoded and deoptimized by several strategies that may involve the introduction of up to thousands of nucleotide (nt) changes without affecting amino acid (aa) coding. The synonymous recoding strategies that have been applied to RNA viruses include: deoptimization of codon or codon-pair usage, which may reduce protein expression among other effects; increased content of immunomodulatory CpG and UpA RNA, which increase immune responses and thereby restrict viral replication; and substitution of serine and leucine codons with synonymous codons for which single-nt substitutions can yield nonsense codons, thus limiting evolutionary potential. This can reduce pathogen fitness and create potential live-attenuated vaccines that may have improved properties. The combined approach of genome recoding, synthetic biology, and reverse genetics offers several advantages for the generation of attenuated RNA viruses. First, synonymous recoding involves many mutations, which should reduce the rate and magnitude of de-attenuation. Second, increasing the amount of recoding can provide increased attenuation. Third, because there are no changes at the aa level, all of the relevant epitopes should be expressed. Fourth, attenuation frequently does not compromise immunogenicity, suggesting that the recoded viruses have increased immunogenicity per infectious particle. Synonymous deoptimization approaches have been applied to two important human viral pathogens, namely respiratory syncytial virus (RSV) and influenza A virus (IAV). This manuscript will briefly review the use of these different methods of synonymous recoding to generate attenuated RSV and IAV strains. It also will review the characterization of these vaccine candidates *in vitro* and in animal models, and describe several surprising findings with respect to phenotypic and genetic instability of some of these candidates.

## Introduction

The availability and affordability of large-scale commercial DNA synthesis opened the field of synthetic biology (1, 2). This technological advance allowed, in 2002, the rescue of an infectious poliovirus entirely from synthetic DNA (3). During the following years, synthetic biology and reverse genetics were combined to design and rescue viruses with extensive targeted modifications. This resulted, in 2006, in the rescue of poliovirus strains with extensive codon deoptimization (CD) (4, 5). This exemplifies the approach of synonymous genome recoding, in which one or more ORFs of a microbial pathogen are modified at the nt level without altering coding at the aa level. Subsequently, synonymous genome recoding has been widely applied to reduce pathogen fitness and create potential live-attenuated vaccines.

Deoptimization by synonymous genome recoding offers several advantages for viral vaccine design. Genomes of recoded viruses may contain up to thousands of synonymous nucleotide mutations in one or several ORFs. Many of these likely contribute to attenuation, in aggregate this large number should impose a significant barrier against reversion to virulence, because any single-site reversion likely would yield only a small amount of de-attenuation (6–8). In principle, the level of attenuation can be modulated by adjusting the number of introduced mutations. Recoded vaccine candidates encode all viral proteins with the same aa sequence as the wt parent, and thus should induce innate, humoral, and cell-mediated responses against the same array of epitopes. Recoded viruses also can contain an increased number of CpG and UpA RNA dinucleotides that may increase host immune responses that restrict the virus and provide greater efficacy. Because synonymous recoding does not involve the lengthy development of specific attenuating mutations, it provides an expedited means of developing attenuated strains of a known or newly emerging pathogen.

Synonymous genome recoding has been applied to two important human respiratory viruses with negative-sense RNA genomes, namely respiratory syncytial virus (RSV) and influenza virus type A (IAV). RSV belongs to the *Pneumoviridae* family and is the most important viral agent of severe respiratory illness in infants and young children worldwide, and also is an important cause of respiratory illness in the frail elderly. Vaccines or antiviral drugs suitable for routine use are not yet available. A live-attenuated vaccine is a strategy of choice for the pediatric population because it is free of RSV disease enhancement that is associated with inactivated and subunit RSV vaccines in RSV-naïve recipients. The RSV genome consists of a single non-segmented negative-sense 15.2 kb RNA, containing 10 genes in the order 3′-NS1-NS2-N-P-M-SH-G-F-M2-L-5′. The M2 mRNA encodes two separate proteins, M2-1 and M2-2, from overlapping ORFs.

IAV belongs to the *Orthomyxoviridae* family, and contains eight RNA genome segments, each encoding one or two proteins: segment 1, PB2; 2, PB1 and PB1-F2; 3, PA, and PA-X protein; 4, HA; 5, NP; 6, NA; 7, M1, and M2; 8, NS1. Antigenic change of IAV is driven by point mutations in the HA and NA proteins as well as segment reassortment. Three types of vaccines are currently licensed for IAV: inactivated, live-attenuated, and recombinant HA protein. This review describes the current strategies of synonymous genome recoding used to generate attenuated RSV and IAV viruses and the characterization *in vitro* and *in vivo* of the resulting vaccine candidates.

## Four Strategies for Synonymous Genome Recoding

The four approaches used to deoptimize the different strains of IAV and RSV and the resulting number of silent nt mutations that have been introduced in these viruses ORFs are summarized in Table 1.

**Table 1:** Attenuation of influenza and respiratory syncytial virus by synonymous genome recoding.

**Virus**	**Deoptimization strategy**	**Virus strain**	**Gene(s) deoptimized**	**Number of silent mutations^j^**	**Main results**	**References**
IAV	CD^a^	Seasonal H1N1	PB2, PB1, PA, HA, NP, NA, M, NS^b^	62, 77, 65, 46, 31, 47, 27, 18	No effect on protein expression *in vitro* Virus attenuated in mammalian cells and in mice Immunogenicity in mice equivalent to wt	(9)
		PR8 H1N1	NS	135	Reduced NS1 and NEP protein expression *in vitro* Reduced virus replication *in vitro* Virus attenuated in mice Immunogenicity in mice equivalent to wt	(10)
	CPD	PR8 H1N1	NP, HA, NA, PB1^c^	314, 353, 265, 236	Reduced protein expression of CPD ORFs *in vitro* Reduced virus replication *in vitro* Viruses attenuated in mice Immunogenicity equivalent to or higher than wt	(11, 12)
		2009 pH1N1^d^	HA, NA^e^	346, 293	Reduced rate of replication *in vitro* Final titers in the lung of ferrets equivalent to wt	(13)
	Increasing CpG or UpA content	PR8 H1N1	NP	86 (CpG-high virus), 73 (UpA-high virus)	Reduced virus replication *in vitro* Virus attenuated in mice Immunogenicity equivalent to wt	(14)
	Mutations in ser and leu codons^f^	2009 pH1N1	HA, PA^g^	94, 111	No effect on virus replication *in vitro* Virus attenuated in mice Immunogenicity equivalent to wt	(15)
RSV	CD	A2	NS1, NS2, G^h^	84, 82, Not indicated	Reduced protein expression of CD genes *in vitro* Reduced virus replication *in vitro* Virus attenuated in mice or cotton rats Immunogenicity equivalent to or higher than wt	(16, 17)
	CPD	A2	NS1, NS2, N, P, M, SH, G, F, L^i^	65, 60, 241, 143, 163, 23, 197, 422, 1,378	Reduced protein expression of CPD genes Reduced virus replication *in vitro* Viruses attenuated in mice, hamsters, and African green monkeys Immunogenicity equivalent to wt	(18)

a *CD, codon deoptimization; CPD, codon-pair deoptimization*.

b *Recoded individually or in the combination of eight*.

c *Recoded individually and in combinations, notably NP-HA-PB1 (11) and HA-NA (12)*.

d *2009 pandemic (p)H1N1*.

e *Recoded in combination*.

f *Serine and leucine codons recoded into synonymous codons for which some single-nt substitutions result in nonsense codons*.

g *Recoded separately*.

h *Recoded in the combinations NS1-NS2 and NS1-NS2-G*.

i *Recoded in the combinations NS1-NS2-N-P-M-SH; G-F; L; and all genes except M2-1 and M2-2*.

j *Number of silent mutations introduced in each gene, respectively*.

### Codon Deoptimization (CD)

Due to the degeneracy of the genetic code, most amino acids are encoded by more than one nucleotide triplet (synonymous codons). Some codons are used more or less frequently than one would expect based on random chance. This unequal frequency of usage of synonymous codons, referred to as codon bias [CB, (19)], can be found in many organisms including viruses (20, 21). CD involves recoding part or all of one or more ORFs to increase the content of synonymous codons that normally are under-represented in the genome of these organisms.

Several hypotheses that have been proposed to explain CB—as well as the effects of CD—involve mechanisms by which CB might affect protein expression (usually to increase expression), and indeed there is a significant association between CB and translation efficiency in *Escherichia. coli (E. coli)* and *Saccharomyces cerevisiae* (22). One hypothesis is that the codon usage of a virus is adapted to the host tRNA abundance, thereby enhancing viral translation and fitness (21, 23–25). Indeed, in several prokaryotes and unicellular eukaryotes there is a consistent correlation between tRNA abundance and the corresponding codon usage frequency (26). This correlation is more difficult to establish for multicellular eukaryotes. In humans, the tRNA abundance varies widely among different tissues. However, the tRNA abundance was statistically correlated to codon usage of highly expressed genes specific for those tissues (27, 28). Nt assignments involved in mRNA secondary structures, and an avoidance of GC content, also might sometimes contribute to CB (19, 29, 30). Of note, the codon bias of negative strand RNA viruses frequently differs from that of their host. A recent study suggests that this discrepancy is due to constraints of the viral replication machinery; in a VSV minigenome system, the purine/pyrimidine content of viral RNAs affected the stability of the viral nucleocapsids and the RNA synthesis activity of viral polymerase complex (31). CB also has been suggested to occur as a means of regulating protein folding: for example, underrepresented tRNAs can decrease the rate of polypeptide chain elongation and thereby improve the quality of protein folding (32). In addition, selective pressure to reduce the content of CpG and UpA RNA, which appear to stimulate innate and adaptive immune responses that would restrict the virus, may contribute to CB (14).

### Codon-Pair Deoptimization (CPD)

Just as codons may appear more or less frequently than expected, the usage of particular pairs of codons may be more or less frequent than expected which is called “codon pair bias” (CPB) (33). For example, in *E. coli*, two codon pairs encoding Leu-Ala [CTG-GCA and CTG-GCG] are highly overrepresented, while the synonymous codon pair CTG-GCC is under-represented. While this non-randomness was evident for pairs of codons, a bias was much less obvious in control analyses evaluating pairs of non-adjacent codons, and almost absent when pairs were separated by two or three intervening codons (33). CPB is thought to affect translation due to differences in the compatibility of different synonymous pairs of aminoacyl-tRNAs in the translating ribosome (33, 34). Buchan and al. suggested that structural features that regulate tRNA geometry within the ribosome may favor specific codon pairs and thus govern genomic codon pair patterns, driving enhanced translational fidelity and/or rate (35). As with CB, other factors may contribute to CPB, such as selection to reduce the content of CpG and UpA RNA thought to be immunostimulatory (36). CPD is achieved by rearranging synonymous codons to increase the frequency of codon-pairs that typically are under-represented, without changing the overall codon usage or nt content. The first CPD of an RNA virus involved poliovirus, in 2008 (37).

### Increasing the CpG and UpA Content

CpG and UpA RNAs typically are under-represented in RNA virus genomes, presumably due to selective pressure to reduce immune recognition by innate immunity sensors. CPD or CD of a viral genome frequently result in inadvertent increases in the CpG and UpA content of the recoded virus, which may account for the increased immunogenicity per PFU that sometimes is observed (11). For several RNA virus genomes, the content of CpG and UpA was deliberately increased while preserving the natural overall CPB and CB ratios (14, 38–40). The resulting viruses were substantially attenuated yet were as immunogenic as their wt parents, presumably due to greater stimulation of innate immune sensors by CpG and UpA resulting in increased immune responses and restriction of virus replication.

Note that CpG DNA can directly and very efficiently activate B cells but also Natural Killer cells, dendritic cells and monocytes/macrophages through TLR9 stimulation (41). While this is less clear for CpG RNA, synthetic CpG RNAs have been shown to stimulate human monocytes resulting in IL-6 and IL-12 production and costimulatory molecule up-regulation. However, this effect is not mediated through TLR3, 7/8, or 9 and the pathways remain to be defined (42). In addition, during virus replication, large amounts of viral mRNAs or double-stranded intermediates are produced that could be potentially be recognized by sensors of the innate immune response. Whether or not the increased CpG and UpA content in recoded viruses results in increased immunogenicity remains to be determined.

Recently, the host Zinc-finger antiviral protein (ZAP) was shown to inhibit a recoded CpG-rich version of HIV-1 by directly binding to regions of the viral RNA rich in CpG RNA. This suggests that ZAP is part of the cellular system for detecting non-self RNA containing CpG RNA (40).

### Synonymous Serine and Leucine Codon Substitutions That Allow Single-nt Mutations Yielding Nonsense Codons

Serine and leucine codons, which have the highest codon redundancy, can be recoded to increase the use of synonymous codons for which some of the possible single-nt changes result in nonsense mutations (“1-to-Stop” mutations) (15). This reduces the number of mutations at a given serine or leucine codon that can yield fit virus, and thus reduces evolutionary potential and viral fitness. This strategy has been applied to the pathogenic enterovirus Coxsackie B3 as well as to IAV (15).

## Methodology Used to Generate Deoptimized RSV and IAV Vaccine Candidates

**Figure 1:**
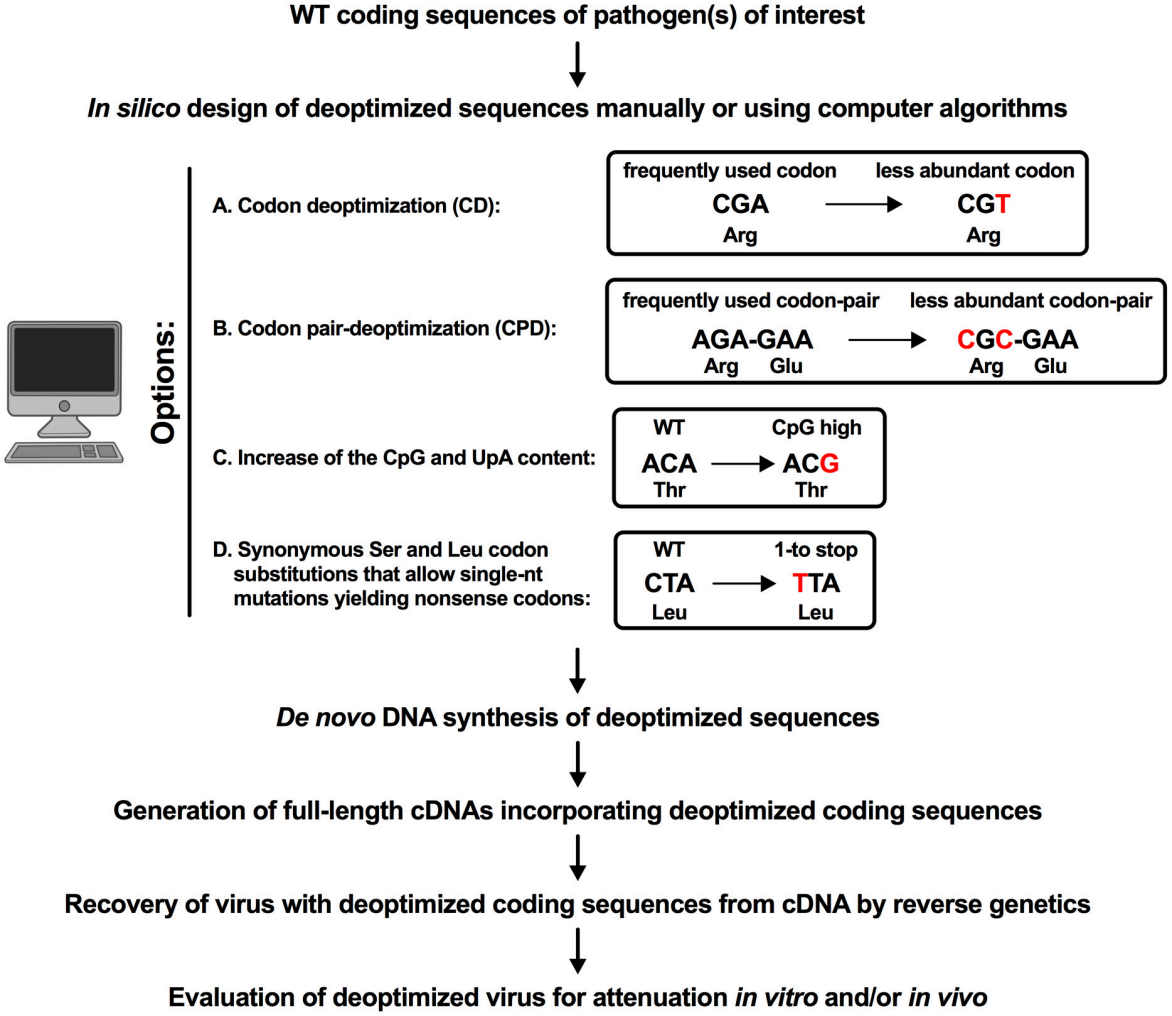
Methodology used to generate genome scale deoptimized RSV or IAV viruses. Four strategies of deoptimization have been used to atttenuate RSV and IAV: **(A)** codon deoptimization (CD), **(B)** codon-pair deoptimization (CPD), **(C)** increase of the CpG and UpA content and **(D)** synonymous Serine and Leucine codon substitutions that allow single-nt mutations yielding non-sense codons. An example is shown for each approach. The synonymous mutations generated by the deoptimization process are indicated in red. In **(A)**, the “A” to “T” mutation resulted in the introduction of an underrepresented Arg codon (10). In **(B)**, the CPD process (37) may yield CpG dinucleotides at codon boundaries that were shown to be significantly suppressed in wt viruses (36). In **(C)**, the program “Sequence Mutate” in the SSE package (43) introduces a synonymous mutation (“A” to “G”) that resulted in the introduction of a CpG motif in the Thr amino acid. Finally in **(D)**, a synonymous mutation “C” to “T” generated a Leu codon “TTA” that by only a single-nucleotide change can generate a stop codon (TAA or TGA) (15).

The methodologies that have been applied to RSV or IAV to generate deoptimized vaccine candidates are described in Figure 1. In all approaches, following identification of the gene(s) or portion(s) of the ORF(s) that will be targeted for deoptimization, silent mutations were first introduced *in silico*, manually (9, 10, 15) or using computer algorithms (11–14, 18), as described below. For each approach, packaging and splicing signals or replication/translation elements were excluded from the deoptimization process.

CD (Figure 1A) of the PR8 NS gene by Nogales and colleagues was done by introducing synonymous mutations to increase the abundance of codons that were under-represented in the natural coding sequences (10). Fan and colleagues used a different approach, as they changed the CB of a seasonal human H1N1 virus to an avian-like IAV CB (9). To do so, the authors first determined the segment specific CBs of the human H1N1 strain and the corresponding avian IAV sequences. This allowed to determine the number of mutations that had to be introduced into the human H1N1 ORFs to change its CB to an avian IAV-like CB. Mutations were randomly distributed in the targeted ORF at sites that were highly conserved at the amino acid sequence level to reduce the possibility of introducing mutations into potential mutational hot spots, or disrupting potential critical RNA signals. The free energy of the resulting RNAs and the dinucleotide usage frequency were unchanged by these modifications.

CPD (Figure 1B) was done using a computer algorithm to enrich a given viral coding sequence for codon-pairs under-represented in a core set of human genes (37). First, the CPB of an ORF is defined by a codon pair score (CPS). This CPS is defined as the natural log of the ratio of the observed over the expected number of occurrences of each codon pair. The expected number of each codon pair was calculated so that it is independent both of the amino acid frequency and the codon bias (33). The CPB for an ORF was then determined as the arithmetic mean of all CPS. The CPD algorithm of Coleman and colleagues (37) shuffles the existing codons of an ORF to generate under represented codon pairs, while preserving the codon bias and the free energy of the folding of the recoded RNA.

The increase of the CpG and UpA content of IAV NP gene (Figure 1C) (14) was completed using the computer algorithm “Sequence Mutate” in the SSE package (43) while maintaining the mononucleotide composition through the introduction of compensatory substitutions elsewhere in the sequence. The CPB was not modified by this process.

Finally, Moratorio and colleagues introduced synonymous mutations that allow single-nt mutations yielding nonsense codons into the HA or PA gene of the 2009 pandemic H1N1 virus only in codons coding for two amino acids with the highest codon redundancy (Leu and Ser) to limit the overall change in nucleotide sequence to <5%. These Leu and Ser mutations did not affect the CB, CPB, CpG content or dinucleotide frequency (Figure 1D) (15).

In every case, the deoptimized sequences were synthetized *de novo* and cloned into plasmids that were used to rescue the deoptimized virus of interest by reverse genetics. The rescued viruses were then evaluated phenotypically *in vitro* and *in vivo*.

## Synonymous Genome Recoding of IAV

### Codon Deoptimization (CD)

CD was applied to the seasonal human H1N1 by converting its codon usage so that it was similar to that observed in avian influenza virus, in order to attenuate the virus for humans without reducing yield in embryonated chicken eggs, the substrate for vaccine production (9). All eight segments were codon-deoptimized alone or in combination. This attenuated the virus in mammalian cells and in mice, whereas replication in embryonated eggs remained comparable to wt. Surprisingly, CD did not affect protein expression (9), illustrating that its effects can be different than predicted.

CD also was applied to the laboratory-passaged H1N1 PR8 strain, involving only the NS gene (10). CD did not affect NS mRNA transcription in MDCK cells but, as expected, did reduce NS protein expression in MDCK and human airway A549 cells, and virus replication was reduced in A549 cells. The CD PR8 virus was attenuated in mice. Both the CD seasonal human H1N1 and the CD PR8 IAV retained their immunogenicity despite attenuation and conferred homologous and heterologous protection against IAV challenge in mice (9, 10).

### Codon-Pair Deoptimization (CPD)

The NP, HA, NA, and PB1 segments of the laboratory-passaged PR8 H1N1 strain were subjected to CPD alone or in several combinations (11, 12). PR8 that contained CPD NP, HA, NA, and PB1 alone or in various combinations replicated to about 10-fold lower titers than wt PR8 on MDCK cells. However, the replication of the PR8/CPD-HA-NA virus in human A549 cells was 1,000-fold lower than wt. As expected, translation of the CPD genes was reduced compared to other genes from the same virus (11). Surprisingly, in case of the CPD NA mRNA, transcription was also reduced, with the underlying mechanism being unclear (12).

Despite overall robust replication *in vitro*, PR8 viruses with various combinations of CPD NP, HA, PB1 genes were attenuated in mice, and the attenuation increased with increasing number of CPD genome segments (11). In mice, the PR8/CPD-NP-HA-PB1virus did not induce any disease symptoms or weight loss and was 3,000-fold reduced for replication compared to wt. In addition, it was a more potent inducer of IAV-specific antibodies than wt, and replication of challenge virus was below the level of detection in 80% of the mice (11). The virus PR8/CPD-HA-NA also was attenuated *in vivo*, replicating to 100- to 1,000-fold lower titers than wt in the lungs of mice, with the NA gene being the major contributor of attenuation (12). This virus also induced a strong antibody response that was equivalent to wt, and it efficiently protected against lethal challenge, with protection being durable for at least 7 months (12).

Since CPD of the HA and NA genes was so highly attenuating for the PR8 virus, the HA, and NA genes of the 2009 pandemic (p)H1N1 strain similarly were subjected to CPD in combination (13). The resulting virus had a reduced rate of replication in MDCK cells, but final titers were similar to those of its pH1N1 parent (13). In ferrets, the CPD-HA-NA virus was non-pathogenic. However, no significant difference in virus titers in the lung at day 3 pi was observed between this virus and its pH1N1 parent, suggesting that additional genes will need to be subjected to CPD to obtain additional attenuation (13). Thus, there can be strain-to-strain variability in the attenuation achieved by CPD.

### Increasing the CpG and UpA Content

Segment 5 (encoding NP) of IAV PR8 was recoded to increase the content of CpG or UpA RNA (14). Replication of the two resulting viruses (CpG-high or UpA-high) was delayed compared to wt in MDCK cells, with final titers that were about 10-fold reduced. Plaque size and infectivity were also reduced. The viruses replicated to 10-fold lower titers than their wt parent in mice, but cytokine production, CD4+, CD8+-T cell and antibody responses were comparable to those induced by wt virus, and the mutants were fully protective against wt challenge. This suggested that CpG- and UpA-high IAV viruses may induce innate and adaptive immune responses disproportionate to their replication phenotypes (14).

### Synonymous Serine and Leucine Codon Substitutions That Allow Single-nt Mutations Yielding Nonsense Codons

Moratorio and colleagues recoded either the HA or PA gene of the 2009 pandemic H1N1 virus to replace serine and leucine codons with synonymous codons for which a number of single-nt substitutions could yield nonsense mutations (15). There was no effect of the recoding on virus replication in MDCK cells. However, both viruses exhibited an increase in nonsense mutations in the mutated genes compared to wt, that significantly reduced viral fitness. In addition, virus replication was reduced 10- to 100-fold in mice. Despite reduced replication, the antibody response was comparable to wt and these viruses induced complete protection against wt virus. The apparent greater immunogenicity per PFU was suggested to be due to immune stimulation by truncated proteins and by adjuvant effects of defective viruses (15). Thus, reducing the evolutionary potential of a virus provides a novel attenuation strategy.

## Synonymous Genome Recoding of RSV

### Codon-Deoptimization (CD)

CD was performed for the ORFs encoding the RSV interferon antagonist non-structural proteins 1 (NS1) and 2 (NS2) (16, 17, 44, 45) and the gene encoding the attachment glycoprotein G (17). As a result of CD, the level of NS1 and NS2 protein was reduced in Vero, BEAS-2B, and Hep-2 cells (16, 44) and the expression of G was reduced in Vero cells (17). CD of NS1 and NS2 did not affect virus replication in Hep-2 and Vero cells, but significantly reduced virus replication in the interferon competent bronchial airway BEAS-2B cells, as well as in differentiated normal human bronchial epithelium (NHBE) cells grown at the air-liquid interface (ALI) (16). The addition of the CD G further reduced virus replication on NHBE/ALI cells, probably due to the role of G in attachment to primary cells (17). Interestingly, while infection of human 293 cells with wt RSV induced a 50% reduction of STAT2 expression, RSV/CD-NS1-NS2 had no effect on STAT2 levels, indicating that this virus had lost the ability to inhibit this aspect of innate immunity. Compared to wt RSV-infected cells, NF-kB activation was reduced in RSV/CD-NS1-NS2- infected cells. The reduced activation of NF-kB by this virus may increase cell apoptosis thus contributing to the attenuated phenotype of this virus (16).

RSV/CD-NS1-NS2 and RSV/CD-NS1-NS2-G replicated to 10-fold lower titers in the lungs of mice compared to wt, but induced significantly higher level of antibodies, and animals were protected against challenge (16). The RSV/CD-NS1-NS2-G virus also was strongly attenuated in the upper and lower respiratory tract of cotton rats, but still induced high levels of antibodies and the vaccinated animals were completely protected against wt challenge (17).

### Codon-Pair Deoptimization (CPD)

Our group studied the effect of genome-scale CPD of RSV (18, 46). Four CPD RSV strains were designed in which one (L), two (G and F), six (NS1, NS2, N, P, M, and SH), and nine (all except M2-1 and M2-2) ORFs were subjected to CPD. All four CPD RSVs were temperature sensitive, which is a novel and unexplained effect, but as one possibility might indicate deficiencies in protein folding resulting from altered translation of CPD ORFs. The viruses grew less efficiently than wt *in vitro* and had reduced mRNA and protein synthesis. CPD of the surface glycoproteins G and F resulted in the strongest reduction in virus replication. The CPD RSVs exhibited a level of attenuation in mice and African Green monkeys comparable with that of two attenuated RSV strains presently in clinical trials (18).

The RSV bearing nine CPD ORFs was phenotypically and genetically stable when subjected to serial passage *in vitro* at progressively increasing temperature. Serial passage at increasing temperature of the RSV bearing the CPD L ORF (Min L) induced a partial loss of temperature-sensitivity and the acquisition of a broad array of mutations that were predominantly missense. Surprisingly, many of the mutations involved ORFs other than L, suggestive of changes affecting protein interactions to compensate for the reduced quantity of L protein. Unexpectedly, each of two compensatory missense mutations in the M2-1 protein had a major effect on restoring viral fitness, which differs from the expectation that individual mutations would have modest effects on viral fitness. The introduction of several of the compensatory mutations identified in the passaged viruses into Min L resulted in increased genetic stability, and the resulting virus was strongly attenuated *in vivo* but was comparable to wt RSV in immunogenicity and protective efficacy, yielding an improved vaccine candidate (46).

## Benefits, Potential Limits, and Future of Synonymous Genome Recoding

Genome scale deoptimization of RNA viruses resulted in the generation of vaccine candidates that in most cases were attenuated *in vitro*, and always attenuated in animal models. While this approach has many advantages that have been described above, the underlying mechanism and resultant effects of the deoptimization still need to be further explored.

Firstly, the extent of deoptimization tolerated by viruses differs widely from virus to virus. For example, while extensive CPD of RSV (up to 9 out the of 11 ORFs CPD) still readily generates a replicating virus, this is not the case for poliovirus or HIV, where extensive CPD or CD did not yield replicating virus. This renders the effect of deoptimization on viral genes hard to predict and implies that, in each case, phenotypes have to be evaluated experimentally. In addition, CD, CPD, and the increase of the CpG and UpA content in some cases resulted in the decrease of the specific infectivity of the recoded viruses. This effect varied depending of the virus and the genes that had been deoptimized and also has to be carefully evaluated on a case-by-case basis.

Most of the approaches described above share common features. For example, the increase of the CpG content was the intended effect in one approach (14), and an also a side effect of CPD. Translation efficiency is expected to be affected both by CD and CPD. With the exception of poliovirus, different deoptimization approaches have not been directly compared side by side using the same genes or portion of genes of a pathogen. This renders the comparison of the efficiency of the different strategies difficult. For poliovirus, both CD and CPD of the same region of the capsid-encoding ORF generated attenuated viruses (5, 37). Interestingly, while both approaches reduced the specific infectivity of the deoptimized viruses, CD reduced the specific infectivity 10-fold more than CPD, suggesting that CD might have a greater effect on the specific infectivity than CPD, at least for poliovirus.

A direct comparison of deoptimized vaccine candidates to those generated by traditional approaches (e.g., biological viruses attenuated by serial passage, recombinant viruses attenuated by gene or codon deletions or non-synonymous attenuating mutations) is also mostly lacking. Pre-clinical evaluation of CPD RSV vaccine candidates showed that the level of restriction of CPD RSVs in African green monkeys was similar to that of two live-attenuated pediatric RSV vaccine candidates presently in clinical trials in infants and young children (18).

Importantly, despite strong restriction of replication *in vivo*, the deoptimized viruses generally induced a strong immune response in vaccinated animals, usually at the level observed with wt virus. As mentioned above, large amounts of viral mRNAs or double-stranded intermediates are produced during virus replication that could be potentially be recognized by the innate immune sensors. It is tempting to speculate that the increased CpG and UpA content in the recoded viruses results in viral mRNAs and/or double double-stranded intermediates with increased immunogenicity. However, this hypothesis would need to be verified. A comprehensive evaluation of the activation and/or proliferation of immune cells (dendritic cells, CD4+, and CD8+ T cells) following stimulation with these viruses would be helpful to elucidate the basis of this strong immunogenicity. In addition, a comprehensive evaluation of the immune response of non-human primates would be informative. Evaluation of the attenuation and immunogenicity of deoptimized vaccine candidates in phase I studies will provide answers on the usefulness of these approaches. Finally, it would also be of interest to complete the reverse experiment by recoding viruses using the most used codon pairs or by further reducing the CpG and UpA content to investigate the resulting effect on virus replication and immunogenicity in animal models.

Overall, the data available to date encourage the further evaluation of these vaccine candidates in clinical trials. However, a more comprehensive understanding is needed of the mechanisms of attenuation conferred by the different strategies of deoptimization. De-attenuation appears to be rare, suggesting that these viruses are genetically stable (4, 5, 16, 17, 37, 47–51). However, an important limitation is that the de-optimized viruses generally have not been subjected to strong selective pressure that would favor the outgrowth of viruses with de-attenuating mutations. In the study in which strong selective pressure was applied to CPD RSV, the virus containing nine CPD ORFs was stable. However, selective pressure on the virus that contained only the CPD L ORF (Min L) resulted in a number of unexpected findings, including de-attenuating mutations outside the CPD ORF that presumably compensated for the low expression of the CPD genes. Importantly, those mutations were used to make a more stable and more immunogenic vaccine candidate. Thus, further studies will be needed to understand escape mechanisms from the restrictions imposed by CPD.

## Conclusion

While genome scale deoptimization of RNA viruses was initiated a decade ago, most of the vaccine candidates generated to date have been evaluated only in animal models. These synonymous recoding strategies may prove useful for developing novel live-attenuated vaccines, such as for pediatric respiratory RSV vaccines as well as for emerging highly pathogenic viruses.

## Author Contributions

All authors listed have made a substantial, direct and intellectual contribution to the work, and approved it for publication.

### Conflict of Interest Statement

CL, PC, and UB are coinventors on a patent application for the development of respiratory syncytial virus (RSV) vaccines by codon pair deoptimization.
